# Exploring the Irish National Folklore Ethnography Database (Dúchas) for Open Data Research on Traditional Medicine Use in Post-Famine Ireland: An Early Example of Citizen Science

**DOI:** 10.3389/fphar.2020.584595

**Published:** 2020-10-29

**Authors:** Aaron Koay, Fiona Shannon, Astrid Sasse, Michael Heinrich, Helen Sheridan

**Affiliations:** ^1^ Trinity College Dublin, NatPro Centre, School of Pharmacy and Pharmaceutical Sciences, Panoz Institute, Dublin, Ireland; ^2^ University College London, School of Pharmacy, London, United Kingdom

**Keywords:** Ireland, ethnography, citizen science, database, schools’ manuscript collection, schools’ folklore scheme, ethnopharmacology

## Abstract

A rich archive of oral and ethnological literature is housed in the National Folklore Collection, in University College Dublin, Ireland. The Schools’ Manuscript Collection is one body of information that contains a wealth of ethnographic material, including that of an ethnomedicinal nature, collected by schoolchildren across Ireland in the 1930s, in an early example of Citizen Science. The collection has been digitized and is available online at Dúchas.ie. Furthermore, there is an on-going and related project, the Meitheal Dúchas.ie Community Transcription project that enables the database to be easily searched, and thus used for research purposes. This study analyses the user interface and functionality of the Dúchas database for ethnomedical research by utilizing probes in the form of plants, within the collection, that have been previously identified as used for medicinal purposes. Limitations and biases associated with both the original collection of the material and the Dúchas database, that impact on the quality and utility of extractable data have been identified, and where possible specific procedures adopted to counteract such limitations. This study provides an insight into; the use of Dúchas.ie for ethnographic research, the use of plants for medicinal purposes in post-famine Ireland and is the first tangible example of Citizen Science in ethnomedical research in Ireland.

## Introduction

Citizen Science is defined as, “the collection and analysis of data relating to the natural world by members of the general public, typically as part of a collaborative project with professional scientists” (OUP, 2020). It is an evolving field of research that is experiencing increased legitimacy with many government institutions and international agencies, such as the United Nations, European Union and environmental protection agencies, all employing it as a research tool (Hecker et al., 2018; Fritz et al., 2019). Schoolchildren are one cohort that can and have participated in citizen science. Recent examples include a study in Austria utilizing schoolchildren to collect ethnobotanical data (Grasser et al., 2016) and the Open Air Laboratories Network, a citizen science initiative that has involved over 4,000 schools in environmental surveys, in the United Kingdom and Ireland (OPAL, 2019). There are also historical examples, such as the Estonian school teacher Gustav Vilbaste who collected ethnobotanical data via school children in the 1930s (Kalle and Soukand, 2014); and the nationwide survey piloted through schools in Ireland in 1937–1939, called the Schools’ Folklore Scheme. The material collected from this initiative, known as the Schools’ Manuscript Collection (SMC), is stored in the National Folklore Collection, University College Dublin, Ireland, has recently been inscribed into the UNESCO Memory of the World Register (UNESCO, 2017), and is the subject of the current study.

The Schools’ Folklore Scheme involved over 5,000 primary schools, over 100,000 pupils between the ages of 11 and 14, and it resulted in approximately 739,000 pages of ethnographic material, providing a huge repository of traditional knowledge in post famine Ireland. The children that participated collected information from older people in their communities on an extensive range of topics on Irish life and culture; including stories, songs, poems, information about how people lived, as well as ethnomedicinal and ethnopharmacological practices and treatments that ranged from plant and natural substances, to religious and symbolic practices (Shannon et al., 2017).

The collection has now been digitized and is available online at https://www.duchas.ie/en. In this paper we explore the use of the Dúchas database, that contains an extensive array of ethnographic material, with a focus on ethnobotanical contributions. There are similar existing databases of digitized archival texts, such as The Welcome Trust online library found at https://wellcomelibrary.org/collections/digital-collections/, the British Library Digitized Manuscripts found at https://www.bl.uk/manuscripts/, HERBA–Historical Botanical database of Estonian Folk Medicine found at http://herba.folklore.ee/, and Manuscripts Online found at https://www.manuscriptsonline.org/. However, there is no directly comparative body of ethnographic information, in terms of how, when and why the material was collected, existing globally.

**FIGURE 1:**
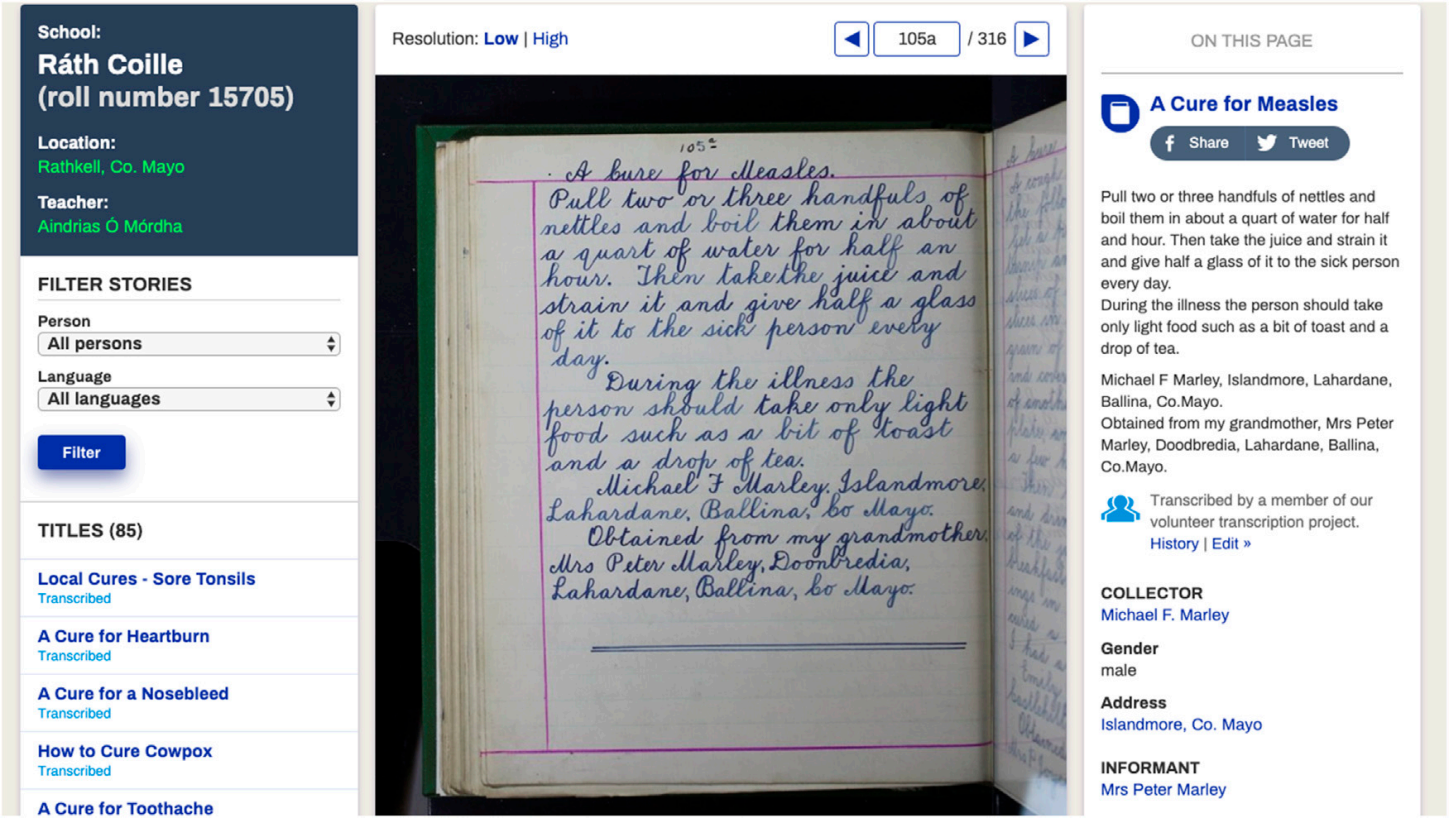
An example of a page of the Schools’ Manuscript Collection that has been transcribed by a volunteer.

An on-going and related project, the Meitheal Dúchas.ie Community Transcription project https://www.duchas.ie/en/meitheal, has enabled a large proportion of the full-text of the collection to be searched through the website’s search engine. This project, launched in 2016, invites users of the site to transcribe, on a voluntary basis, the stories that were collected as part of the Schools' Collection, from scanned images of each page of the collection, to written text (Figure 1). This is an interesting example and opportunity for engagement with citizen science in 21st century Ireland. Easy access to such a huge body of dynamic, diverse and evolving information via the Dúchas.ie website and the Meitheal Community Project, facilitates novel Irish ethnological research on the 1930’s to be conducted efficiently. As the Meitheal project is still on going, the level of transcription has increased since the writing of this paper. To date, there is only one study based on the Dúchas database, that addressed ethical considerations in publishing sensitive material from the collection (Cleircín et al., 2015). There are no studies on the use of the database.

For this analysis probes are used to explore the functionality and utility of the Dúchas database, highlighting its value for international online research. The probes chosen are based on findings from an on-going study on the SMC that has identified the top 10 most highly cited plants used for medicinal purposes in counties Roscommon and Wexford. The study involves transcribing, collating and analyzing all the ethnomedicinal information from the two counties, from the physical collection of manuscripts. In this study, we are utilizing these top 10 plants as probes to illustrate how the Dúchas website can be used to extract, analyze and interpret data therein.

## Methods

### Study Area

**FIGURE 2:**
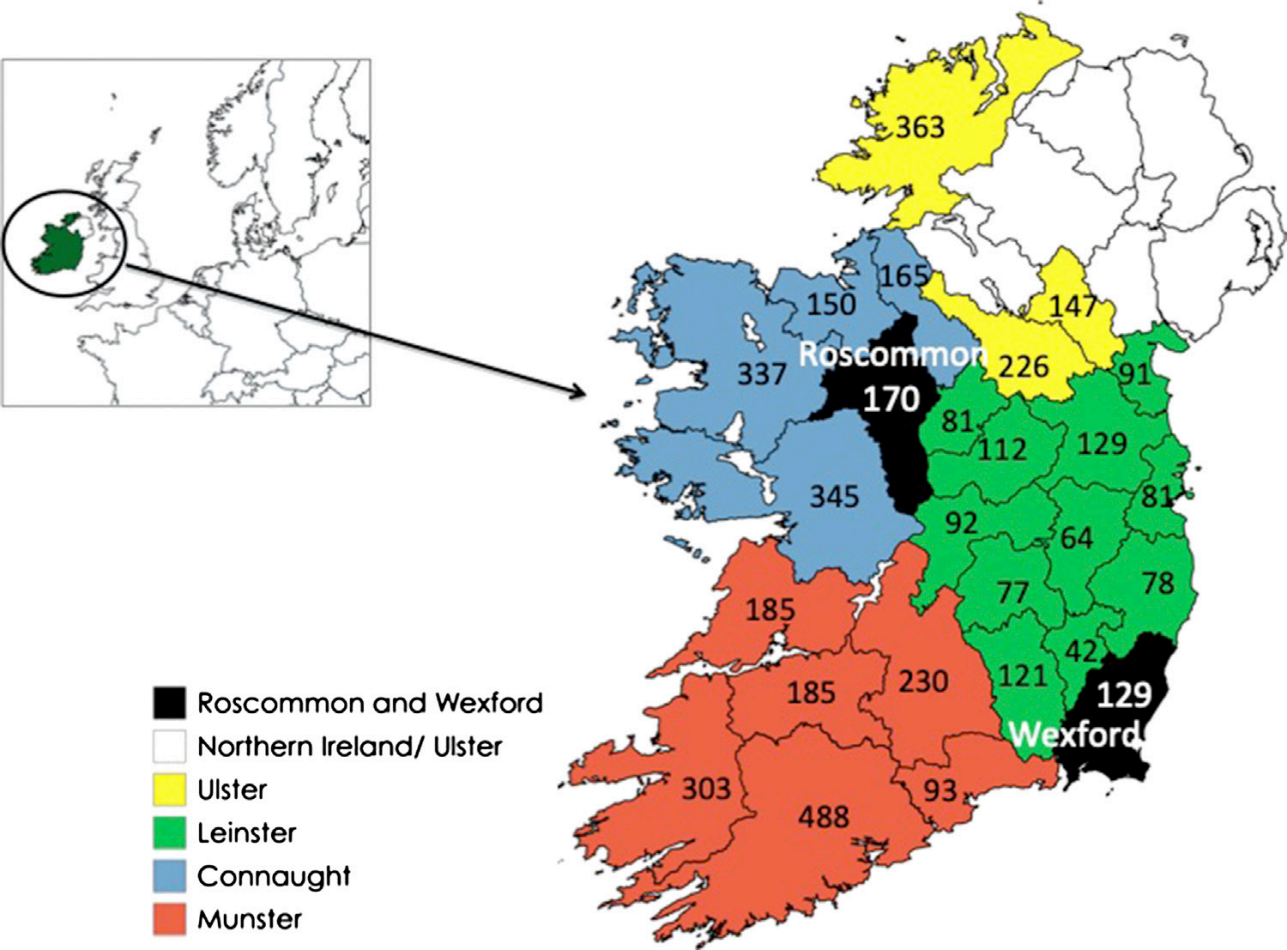
Map of Ireland showing four provinces, counties Roscommon and Wexford and the number of manuscripts submitted to the Schools’ Manuscript Collection from each county.

The area covered for data collection in the 1930s archive related to the 26 counties of the newly founded Republic of Ireland. Analysis of the archival material shows records for each of the counties that took part in the SMC (Figure 2).

### Selection of Probes Used to Explore the Database

Ten plants were selected as “probes” for this analysis based on a recent study of the SMC where the most frequently cited plants from two regions of Ireland were identified (Shannon et al., 2018). This number was reduced to seven (see below) and of these, three plants were prioritized. All the “plant” names that are documented in the SMC are in vernacular form, as the pupils did not have any botanical knowledge. As a result, reference tools such as the Irish plant book “Ireland’s Generous Nature” (Jackson, 2014) and Kew Gardens online checklist www.theplantlist.org were utilized to identify the most probable plant species where possible.

### Dataset Construction

The digitized SMC was accessed via Duchas.ie. The total number of manuscripts in the Republic of Ireland and each county was recorded from Duchas.ie/en/cbes. The vernacular name of each “probe” or plant was entered into the search engine to retrieve the total number of transcripts in the country, which was then refined by county using the in-built filter function on the website. The total transcript counts in the country and in each county were recorded. All the data was retrieved on 24 September 2018. Two map graphs, each depicting the number of manuscripts and retrieved transcripts for the 10 plants in each county of the ROI was created using the “3D Map” function of Microsoft Excel. The dataset was presented in a heatmap and data bars using the “Conditional Formatting” function of Microsoft Excel.

### Data Normalization

The transcript count of each plant in each county was normalized against the number of manuscripts in the respective county. Following that and taking into account the limitations discussed under *Results and Discussion*, the normalization parameter was changed to the total transcript count of the 10 plants in the respective county.

### Data Exclusion

The transcripts retrieved for each plant were scanned for their relevance and those that showed citations in contexts irrelevant to medicinal uses were removed from the dataset. This was done by the “Find” function in the Google Chrome web browser using the “Ctrl + F” command. The vernacular name of the plant of interest was inserted into the “Find” box and the “next” button was used to reveal relevant citations, allowing efficient scanning of the transcripts. As such, tea, potato, and tobacco were completely excluded from this analysis, as a significant portion of their citations did not refer to medicinal treatments. Thus, the normalization parameter was altered and the previously generated heatmap and data bars were updated accordingly. Bar charts presenting the normalized number of transcripts of the seven plants in each province as well as in counties Roscommon and Wexford were generated using Microsoft Excel.

### Data Mining

The number of manuscripts from each county was noted. The top seven plants being used as “probes” were searched within each county and the number of transcripts relating to each plant was identified and then normalized against the total number transcripts of the top seven plants, in the respective counties.

Subsequently, this study focused on the three most highly cited plants, selected as Dandelion, Dock, and Nettle. Their historical and medicinal importance were reviewed through ethnobotanical textbooks and online databases such as Google Scholar and PUBMED. Variations of their vernacular and Latin names were included in the searches. The cited medicinal uses of these three plants were extracted with details including plant parts, preparation methods, route of administration and anecdotes documented.

The excerpts were counted and grouped using the previously established disease classification system’ generated by (Shannon et al., 2018). The system is based on two existing classification systems that have similarities to the material from the Schools’ Folklore Scheme; The International Classification of Primary Care 2nd edition (WICC, 2015), and a system that was established in a 2016 study to analyze a translation of Dioscorides’ De Materia Medica (Staub et al., 2016). Following this, they were further refined into specific treatment conditions (similar conditions that fell within the same disease classification), and then by method of administration. Ambiguities in the terminology used in the transcripts were addressed as discussed in *Results and Discussion*. The data organization procedures were conducted manually with Microsoft Excel. Both the transcript counts for each disease class and each treatment condition were normalized against the total transcript count of the respective plant in the respective county. Pie charts were generated using Microsoft Excel to reveal the normalized percentage of disease classes cited for treatment by each plant in each county. Additionally, a SWOT analysis carried out on the Dúchas database and ethnomedicinal data housed therein.

## Results and Discussion

The Republic of Ireland is made up of four Provinces with 26 counties therein (Figure 2). In the 2018 study, where the plant “probes” for this analysis were obtained, all the ethnopharmacological information in the SMC for two regions in Ireland, counties Wexford and Roscommon were collated and analyzed. These data were accessed manually as the digitized online database that now exists was not complete. A total of 5,224 data entries were extracted from 190 schools (Shannon et al., 2018).

### Duchas.ie: Interface and Functionality

The digitized SMC archive can be viewed online at Duchas.ie, allowing global access with ease to the documentation within. The functionality of the archive is discussed as is presented at the date of access (8 February 2019).

**FIGURE 3:**
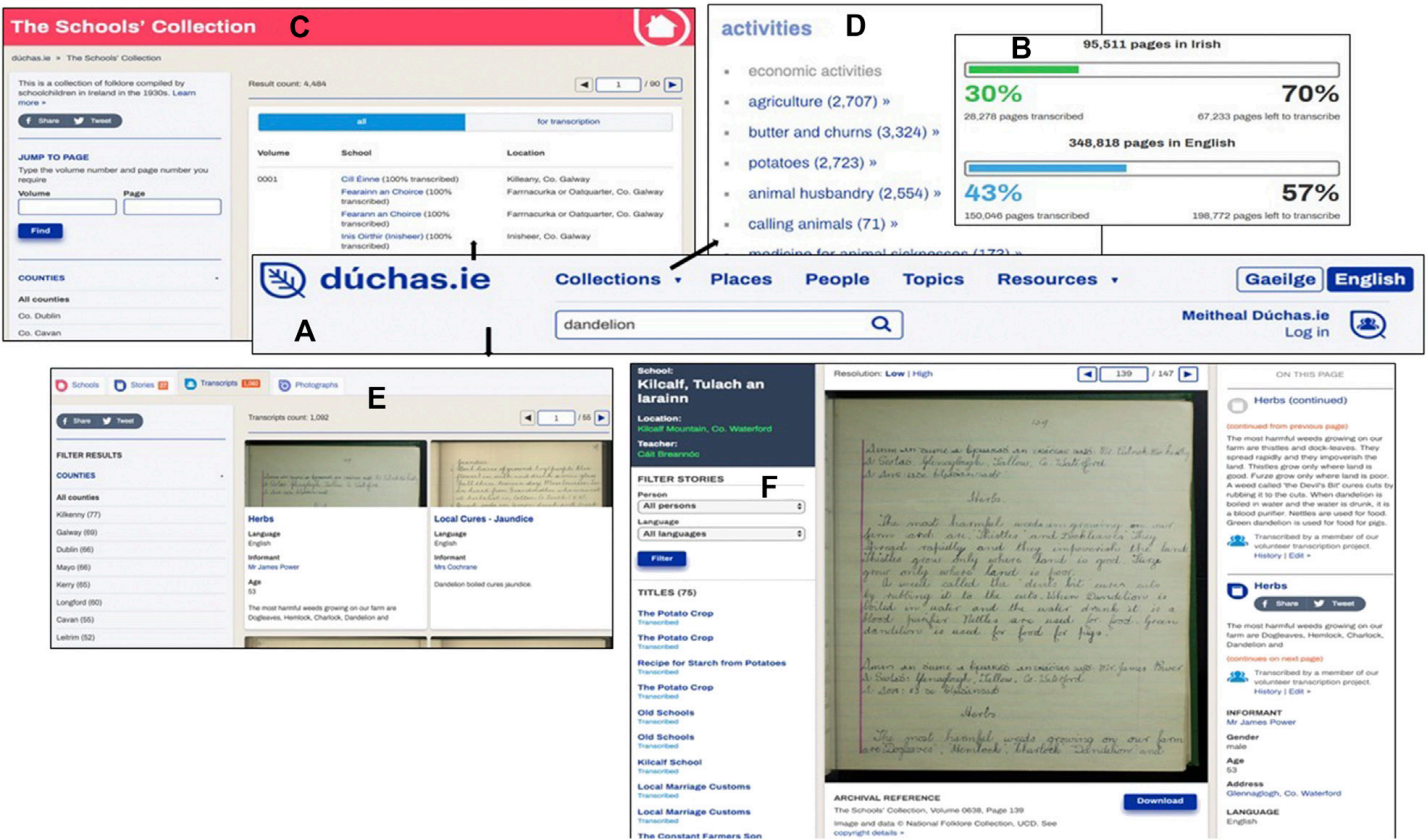
Screenshots showing the interface and functionality of the Dúchas website as accessed on 8th February 2019. **(A)** The search panel and starting point of database navigation located on the main Dúchas homepage and all other pages; **(B)** the transcription rate documented for pages in Irish and English; **(C)** the main page that shows a list of the manuscripts with volume number, school name and transcription rate; **(D)** a list of cataloged topics from transcribed material; **(E)** retrieved transcripts from the search term “dandelion”; and **(F)** details of a retrieved transcript with image of page and transcription.

On the homepage of the website, a panel incorporated with multiple functions is presented in the middle (Figure 3A). The website can be read in either Gaeilge or English and such language preference could be made on the panel. Below the language buttons, there is a link that leads to details about the Meitheal Community Transcription Project; therein the voluntary opportunity to sign up and participate is also described. As of 8 February 2019, only 30% of the Irish pages and 43% of the English pages have been transcribed (Figure 3B).

Hovering the mouse over the “Collections” button will reveal the three collections stored in this digital archive, one of which is the SMC. Of note, the SMC is termed “The Schools” Collection’ in the digital archive but the discrepancy is not meaningful (Figure 3C). Details of the manuscripts, such as volume number, school, location, and transcription rate can be accessed here. The filter function on the left panel allows easy and specific access to the materials according to the volume and page number or the county of interest.

Moreover, thematic browsing of the collections is available by clicking the “Topics” button (Figure 3D). However, free-text searching is not in-built within each topic. To illustrate, all transcripts indexed “folklore medicine” can be accessed and viewed but searching a keyword of interest, such as “dandelion,” within those retrieved transcripts are not an available feature.

Searching text using the toolbar on the homepage panel will generate results that are automatically sorted by “Schools,” “Stories,” “Transcripts,” and “Photographs” (Figure 3E). The number of results generated is also shown. For the purpose of the study, the functionality of the “Transcripts” was further explored as transcripts citing the relevant keyword, which can also be filtered by county using the left panel, are revealed here. After the search term has been inputted, e.g., “dandelion,” part of the scanned pages as well as details including the title, language, informant, age and excerpt of the retrieved transcripts are shown where available.

Clicking on the title can reveal more information about the transcript of interest (Figure 3F). The school, location and teacher of the student are shown on the left panel. The transcript can be read with either low or high resolution and the image is automatically magnified when the mouse is hovered over. If the transcript has been transcribed, the text can be read on the right panel. The history of the amendment of the transcript can be viewed and the user can make any corrections using the right panel. Furthermore, the image of the transcript can be downloaded, and the suggested reference is provided below the image. A link to the instructions regarding the problem-reporting system and the “Notice and Action” procedure in place to counteract inappropriate use of the materials is also available.

Notably, social media sharing functions are incorporated throughout the digital archive, allowing the reader to conveniently share the archival materials on social media platforms like Facebook and Twitter.

### Transcriptional Variations Within the Digitized Data: Differences by County

**FIGURE 4:**
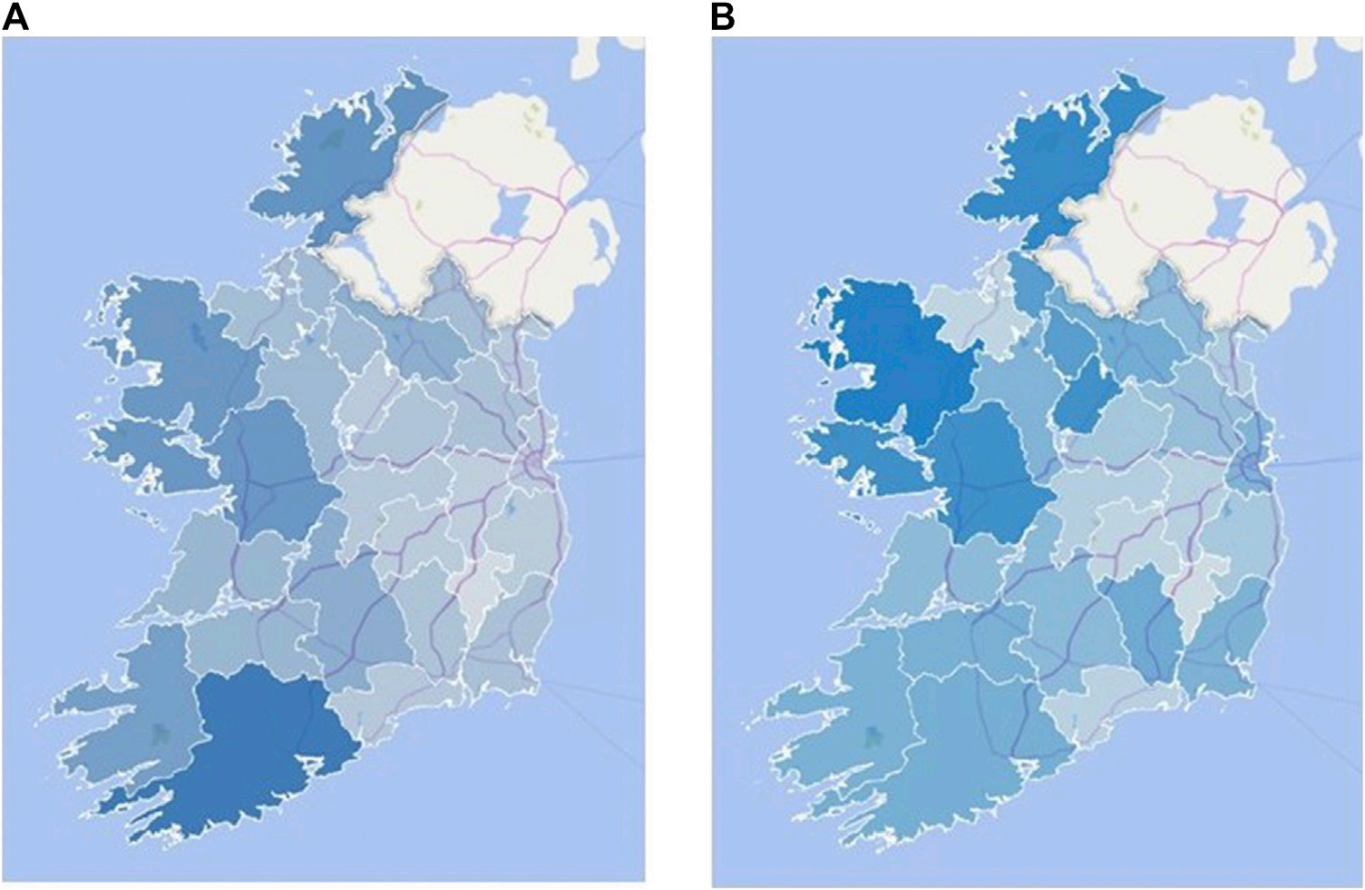
Map graphs of Ireland **(A)** the number of digitalized manuscripts (42–488), **(B)** the number of retrieved transcripts citing the top 10 plants (139–848) in each county of the Republic of Ireland. The intensity of the blue scale correlates with the actual number of manuscripts. The graphs were created using the “3D Map” function of Microsoft Excel.

The maps depicted in Figures 4A,B reveal that there is a different extent of disparity between the amount of manuscripts from each county and the amount of manuscripts that have been transcribed through the Meitheal project and that include the top 10 plants listed in Table 1, for most counties. For instance, notwithstanding that county Cork shows the greatest density of digitized manuscripts (darkest blue color in Figure 4A), its total number of retrieved transcripts citing the top 10 plants, is in the mid-range in the country, where the converse is true for county Mayo with the greatest density of retrieved transcripts, i.e., darkest blue color in Figure 4B. On the other hand, a number of counties show comparable quantities, i.e., similar shade of blue, in both, the number of digitized manuscripts and retrieved manuscripts citing the top 10 plants (Figures 4A,B).

**TABLE 1:** Most highly cited plants documented in ongoing study of the ethnomedicinal entries from Counties Wexford and Roscommon in the Schools’ Manuscript Collection (Shannon et al., 2018).

No	Common name	Most probable species	Plant family
1	Dandelion	*Taraxacum officinale* (L.) Weber ex F.H.Wigg	Asteraceae
2	Dock	*Rumex* species	Polygonaceae
3	Nettle	*Urtica dioica* L.	Urticaceae
4	Potato	*Solanum tuberosum* L.	Solanaceae
5	Tea	*Camellia sinensis* (L.) Kuntze	Theaceae
6	Ivy	*Hedera helix* L. or *Hedera hibernica* (G.Kirchn.) carrière	Araliaceae
7	Garlic	*Allium sativum* L.	Liliaceae
8	Comfrey	*Symphytum officinale* L.	Boraginaceae
9	Marshmallow	*Althaea officinalis* L.	Malvaceae
10	Tobacco	*Nicotiana tabacum* L.	Solanaceae

**FIGURE 5:**
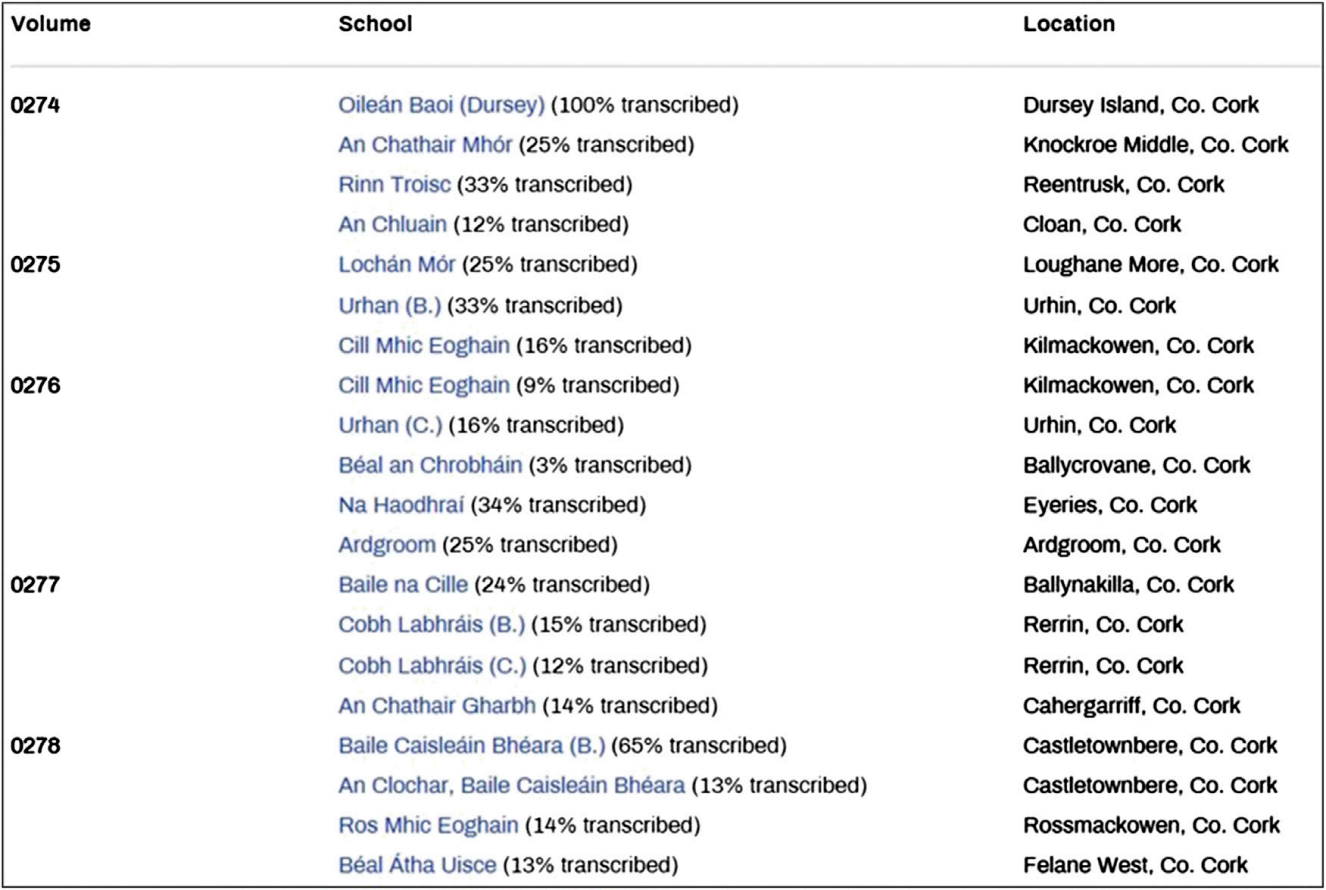
Screenshot of the transcription rates of part of the Schools Manuscript Collection in county Cork taken from https://www.duchas.ie/en/cbes/CO?p=1&o=Vol on 27 January 2019.

A plausible reason for the disparity could be different transcription rates in each county; not all digitized SMC manuscripts have been transcribed and are therefore retrievable from the in-built full-text search engine. To exemplify, despite possessing the highest number of manuscripts (Figure 4A), the relatively low transcription rate in county Cork as illustrated in Figure 5 rendered the retrieved data shown in Figure 4B incomplete and hence inaccurate. However, it should be emphasized that the volume of manuscripts is not a parameter to estimate the transcripts and prevalence of the plants. Notably, low transcription rates were also observed in counties in which numbers of manuscripts and retrieved transcripts are similar, i.e., light blue shade in all maps shown in Figures 4A,B.

### Biases in Data Collection

The methodology utilized in the Schools’ Folklore Scheme involving teachers and schoolchildren has resulted in potential biases. The teachers that participated in the scheme received a booklet of instructions that included hundreds of questions and suggestions for compiling information on a wide range of folkloristic and ethnological themes, that were arranged under 55 different headings. The booklet is available at https://www.duchas.ie/download/17.01.26-irish-folklore-and-tradition.pdf. Based on the information in the booklet, the teacher. From these suggestions, they decided on what specific questions and topics they wanted their pupils to collect information on. Furthermore, when the schoolchildren brought the information that they collected to the teacher, he or she decided what data went into the final manuscript that was submitted.

Thus, although there were standard instructions, the flexibility and freedom in data collection and recording inevitably allowed personal preference to influence the nature and volume of information collected. For instance, the high density of transcripts relevant to folklore medicine in a county might not reflect the richness and wide adoption by the population but rather greater encouragement to the schoolchildren in collecting such information by the teachers due to their biased preference. Therefore, due to this intrinsic limitation in the collection procedure, the rich and comprehensive documentations cannot accurately reflect the real breadth and depth of the Irish ethnological knowledge in each county.

#### Two-step Data Normalization Procedures

In order to counteract the above limitations, two data normalization procedures were investigated. The retrieved number of transcripts for each plant were normalized against:

1. The total number of manuscripts in the respective county.
2. The total number of retrieved transcripts for the 10 plants in the respective county.

Heatmaps were generated to illustrate the density distribution of total number of manuscripts and of citations of the plants across the country to facilitate scrutiny of the dataset.

Neither of the limitations could be fully addressed by the first normalization parameter. If the SMC was completely transcribed, this normalization procedure would have been capable of diminishing the disparity in the number of manuscripts in each county. However, there are differences in the transcription rates in each county (e.g., county Cork in Figure 5). Likewise, the biases as a result of the methodology utilized in the collection of material will not be captured by such normalization procedure either.

Alternatively, the second normalization parameter could address the issue of transcription variations, by normalizing the data of each plant to the transcripts for the 10 combined plants in all counties. However, this analysis was unable to mitigate the issues pertaining to biases in collection methodology.

#### Data Exclusion to Increase Dataset Objectivity

In the initial heatmap generated following the second normalization procedure, tea and potato were dominant among all plants in the retrieved transcripts (data not shown). This is since the archive contains a wealth of ethnological information and not solely medicinal uses. As tea and potato are significant plants in areas such as nutrition, agriculture, history, anecdotes and arts and crafts, they were frequently cited in those contexts. Thus, they were excluded from the study.

**FIGURE 6:**
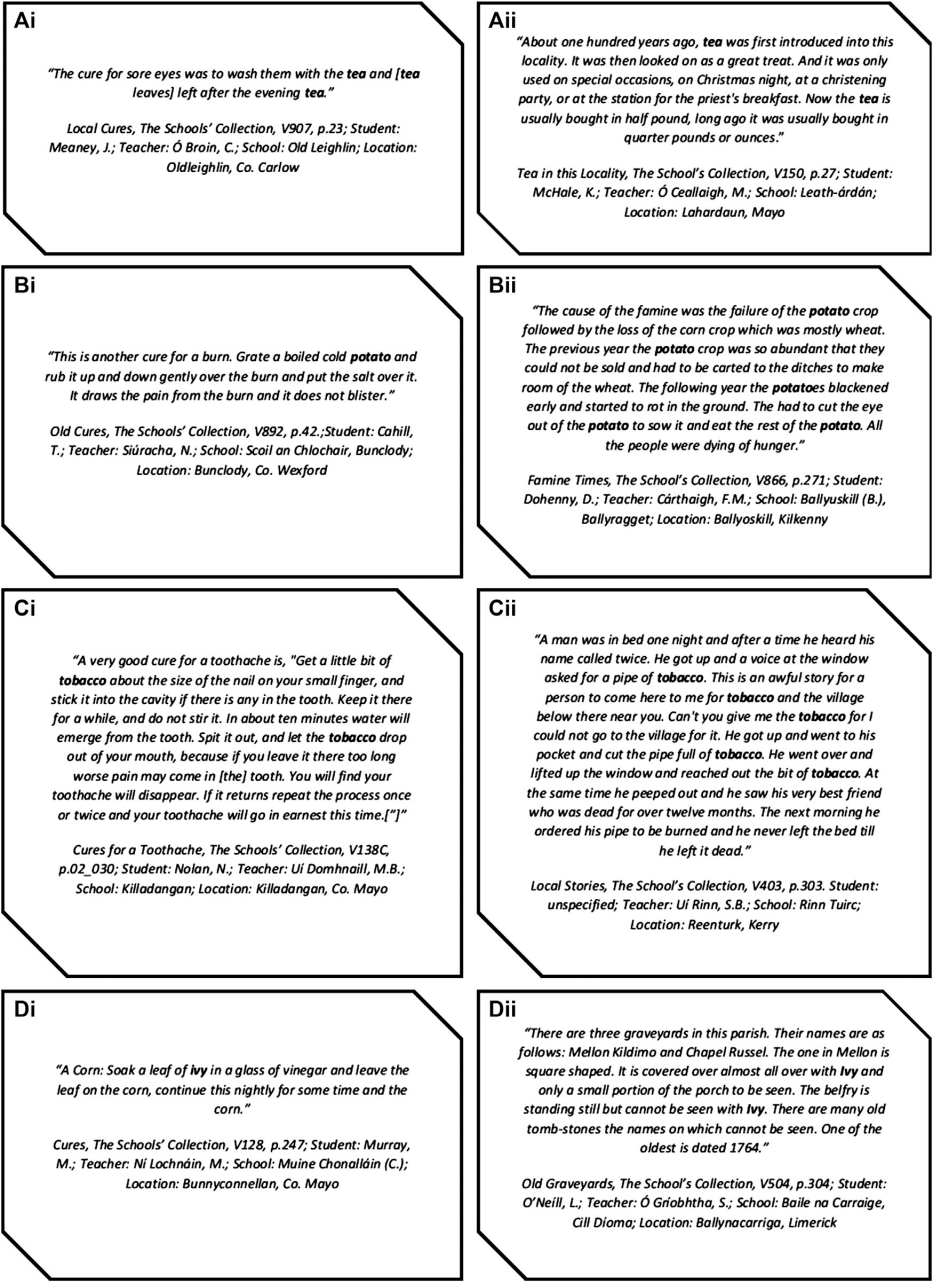
Excerpts of transcripts citing **(A)** tea, **(B)** potato, **(C)** tobacco, and **(D)** ivy in contexts **(i)** relevant and **(ii)** irrelevant to medicinal uses. During the data exclusion procedure, retrieved transcripts were scanned for the citations using the “Find” function of Google Chrome web browser.

**FIGURE 7:**
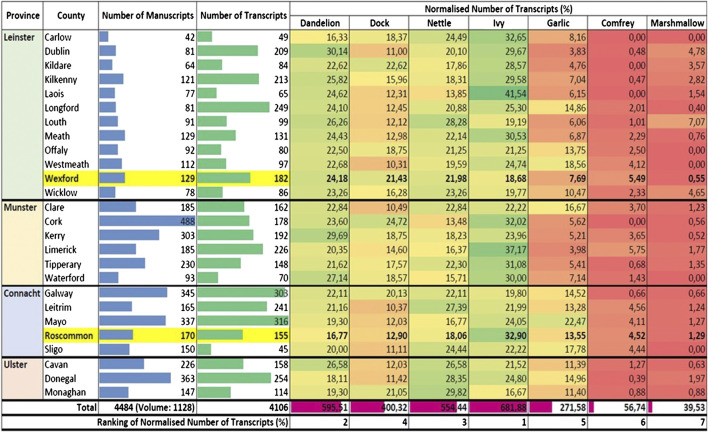
Heatmap depicting the normalized number of transcripts (%) of each plant in each county (green to red; highest to lowest); data bars depicting the number of manuscripts (blue), the number of transcripts of the seven plants (green) and the total normalized number of transcripts (%) of each plant. The normalization parameter is the number of transcripts of the seven plants in the respective county. The heatmap and data bars were created using the “Conditional Formatting” function of Microsoft Excel.

Examples of both medicinal and non-medicinal citation of several plants are presented in Figure 6. Of note, the examples of medicinal uses of the plants were retrieved using the search full website functionality of Google search engine rather than the in-built “Find” function of the database, which carries several limitations; please refer to *Limitations*. Despite indexation of the transcripts, searching full text within a topic, e.g., “folk medicine,” is not yet an available function of the digitized archive. The vast number of transcripts also prevented manual exclusion of irrelevant transcripts. The inclusion of such transcripts risks skewing the data and leads to inaccurate representation of the recorded knowledge. In order the attenuate this limitation, the plants that showed significant citations in contexts other than medicinal uses, namely tea, potato and tobacco, were removed from the dataset. As such, the heatmap was updated accordingly (Figure 7).

The normalized number of transcripts citing each plant in each of the 26 counties of the country is illustrated in Figure 2. Notably, the ranking of the citations of the cited plants is almost in accordance with that of medicinal uses established by Shannon et al. (2018). Ivy is the most frequently cited plant in the heatmap, followed by dandelion, nettle, dock, garlic, comfrey, and then marshmallow. The high citation of ivy can be attributed to it being mentioned widely in several different contexts such as architecture (Figure 6Dii). However, as the number of transcripts of ivy in medicinal uses was found to be significant during the data exclusion procedure, its status remains unchanged in this study. Unexpectedly, garlic shows a relatively low number of citations despite being a historically important plant that was used globally as a food and medicine throughout history and is the focus of significant biochemical and pharmacological research (Corzo-Martínez et al., 2007; Farag et al., 2017). Moreover, the normalized numbers of transcripts of comfrey and marshmallow are significantly lower than all the other plants; with the latter being the least cited plant; this can be explained by the limitations, with perhaps the incomplete transcription being the main one.

**FIGURE 8:**
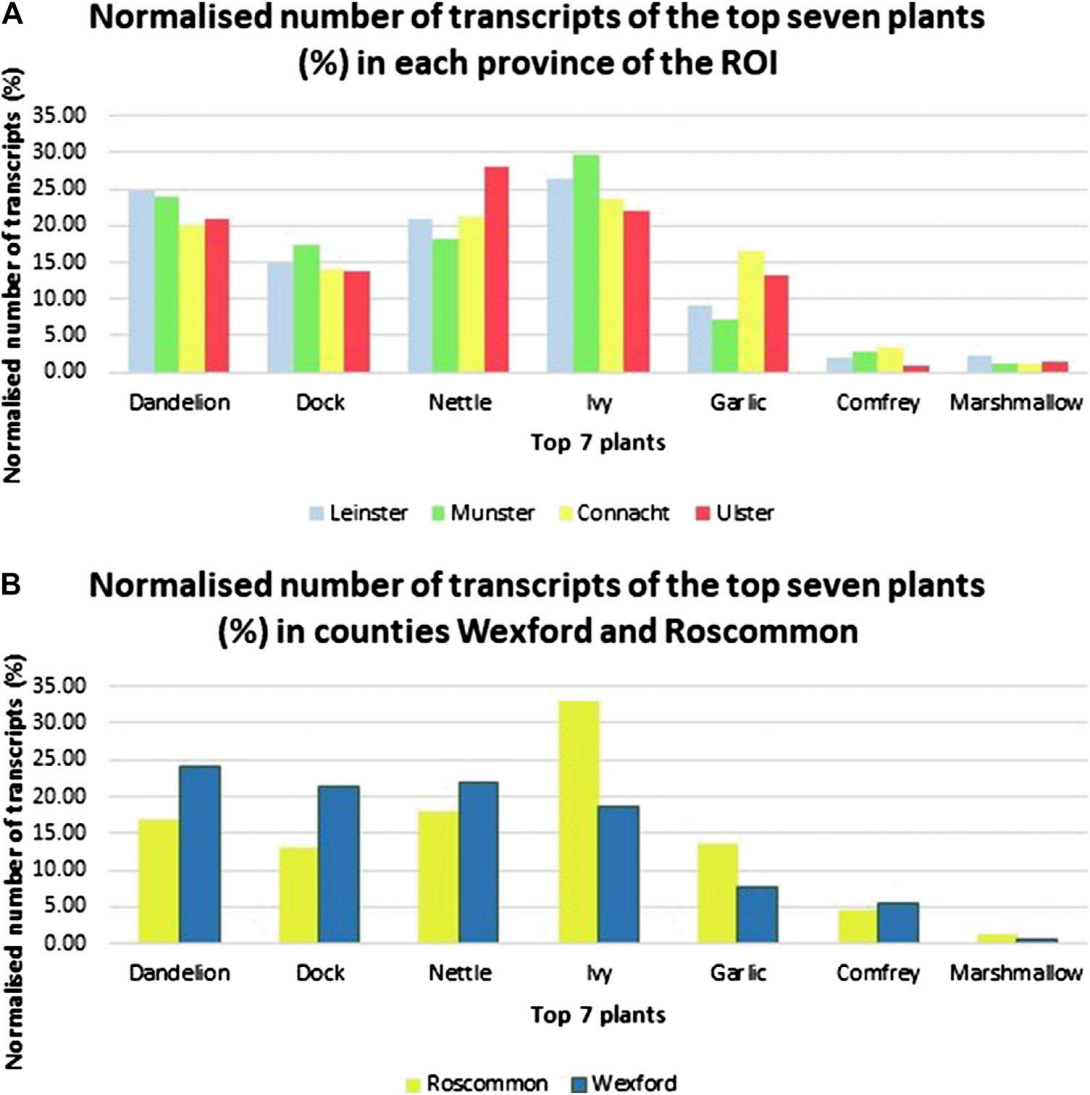
Normalized number of transcripts of the top seven plants (%) in **(A)** each province of the Republic of Ireland and **(B)** counties Roscommon and Wexford. The graphs were created using Microsoft Excel.

Data after filtering by province is reflected in Figure 8A. The graph depicts the variation of the number of normalized transcripts in each province. For example, there are significantly higher citations of garlic in Connaught and Ulster than the other part of the country. The data was subsequently filtered to the two counties Roscommon and Wexford (Figure 8B) as the “probes” for this study were based on the results from analysis of these two counties (Shannon et al., 2018). Wexford shows a higher normalized number of citations than Roscommon in more plants, namely Dandelion, Dock, Nettle, and Comfrey. Of note, the order in which the seven plants rank in terms of the number of citations in the two counties is the same as that in the whole country and that established by the 2018 study (Shannon et al., 2018).

### Probing the Digital Archive Using the Top Three Plants

As a result of using 10 plants as “probes” in our analysis of the Dúchas.ie website we identified the Dandelion, Dock and Nettle as the most statistically significant plants. All three species are commonly found throughout Ireland as well as globally. Their historical and traditional use supports our findings (Jackson, 2014).

Dandelion: Over 2,500 species of “Dandelion” *Taraxacum* spp. have been documented globally (Martinez et al., 2015). However, for the purpose of this study the “Dandelion” recorded in the SMC has been identified as *Taraxacum officinale*, as this is the most prolific species found throughout Ireland and there is significant historical literature to support its medicinal use (Moloney, 1919; Logan, 1972; Logan, 1981; Allen and Hatfield, 2004; Jackson, 2014; Shannon et al., 2017). Some of the recommendations documented in the SMC included using the white sap from the stem for external use on warts, boiling the roots and or leaves to make an internal concoction for a “bad liver,” “the stomach,” “anemia” or “consumption.” There is extensive evidence of the global use of *T. officinale* throughout history, dating back to the 10th and 11th century Arabian medicine (Martinez et al., 2015). In the 16th century, the German physician and botanist Leonhard Fuchs documented its use for complaints such as gout, diarrhea, blister, spleen and liver complaints, and Culpeper (1,653) also recommended the roots and leaves for the liver and spleen (Schütz et al., 2006). In recent years, The European Medicine Agency has recognized *T. officinale* root and herb, based on “well-established use” as a treatment for digestive and urinary complaints (European Medicines Agency, 2009; Shannon et al., 2017). Additionally, a research initiative called “The Dandelion Root Project” based in The University of Windsor, Canada has been doing extensive research on the plant root and its potential anticancer effect. As a result, it has now successfully brought the first natural extract Dandelion Root Extract with anticancer efficacy to phase one clinical trial (Pandey et al*.*, 2011; Dandelion Root Project, 2019).

Dock: The “Dock” plant described in the SMC is more difficult to identify. As there are several different *Rumex* species in Ireland that are historically referred to as dock for various medicinal purposes, there is no way of establishing what species of *Rumex* the contributors are referring to in the SMC, or if they are all referring to the same. The two most used species are *Rumex obtusifolius and R. crispus*. For the purpose of this study we have labeled all references to “Dock” as *Rumex* spp. Most entries recommend *Rumex* spp. for treatment of a “nettle sting.” *R. obtusifolius* is the species widely known for the ability to ease the sting of a nettle (Jackson, 2014). However, the nature of this analysis did not allow for interrogating the data to such an extent to extract the different uses for different species of *Rumex*. *Rumex* spp. is indigenous to Europe and *R. obtusifolius* has been introduced to many other parts of the world, including Africa, and North and South America (Cavers and Harper, 1964; Shannon et al., 2017).

***Nettle***: The “Nettle” plant is found abundantly throughout Ireland (Jackson, 2014). It is generally identified as *Urtica dioica* although there is also a small nettle variety, *Urtica urens* that is very similar. In the SMC, it was mostly recommended as a “spring tonic,” or good for “blood.” There is a long history of use of Nettle in Ireland as a tonic (Allen and Hatfield, 2004; Dolan, 2007; Ballard, 2009; Jackson, 2014) Dioscorides describes how when nettle leaves are cooked together with mussels they soothes the stomach, drive away flatulence and propel the urine (Leonti et al., 2009). Additionally, The European Medicines Agency has listed the use of *U. dioica* L., *U. urens* L., their hybrids or their mixtures, radix, based on well-established use as a herbal medicinal product for the relief of lower urinary tract symptoms related to benign prostatic hyperplasia (European Medicines Agency, 2012).

#### Distribution of Dandelion, Dock and Nettle in Ireland: Pre-1930–1939

The occurrence and distribution of the three plants in Ireland were investigated using the Botanical Society of Britain and Ireland distribution maps available at https://bsbi.org/maps. The maps illustrate sparse records of all three plants in all the island of Ireland (data not shown). Nevertheless, the high extent of similarity in the distribution of each plant among the generated maps, such as large clusters of records after 2010 in the southeast, might prove the database biased and incomplete. Moreover, the number of occurrence records from pre-1930 to 1939 (data not shown), which is of the interest of this study, was limited. Hence, the validity of the Botanical Society of Britain and Ireland distribution maps as an investigational tool in ethnopharmacology research in Ireland during the period of interest might be questionable. However, it is plausible that the ubiquitous occurrence of the three plants as illustrated by the map graphs suggests their infiltration into the folk culture of the Irish population.

#### Medicinal Uses of Dandelion, Dock, and Nettle in Counties Roscommon and Wexford

**FIGURE 9:**
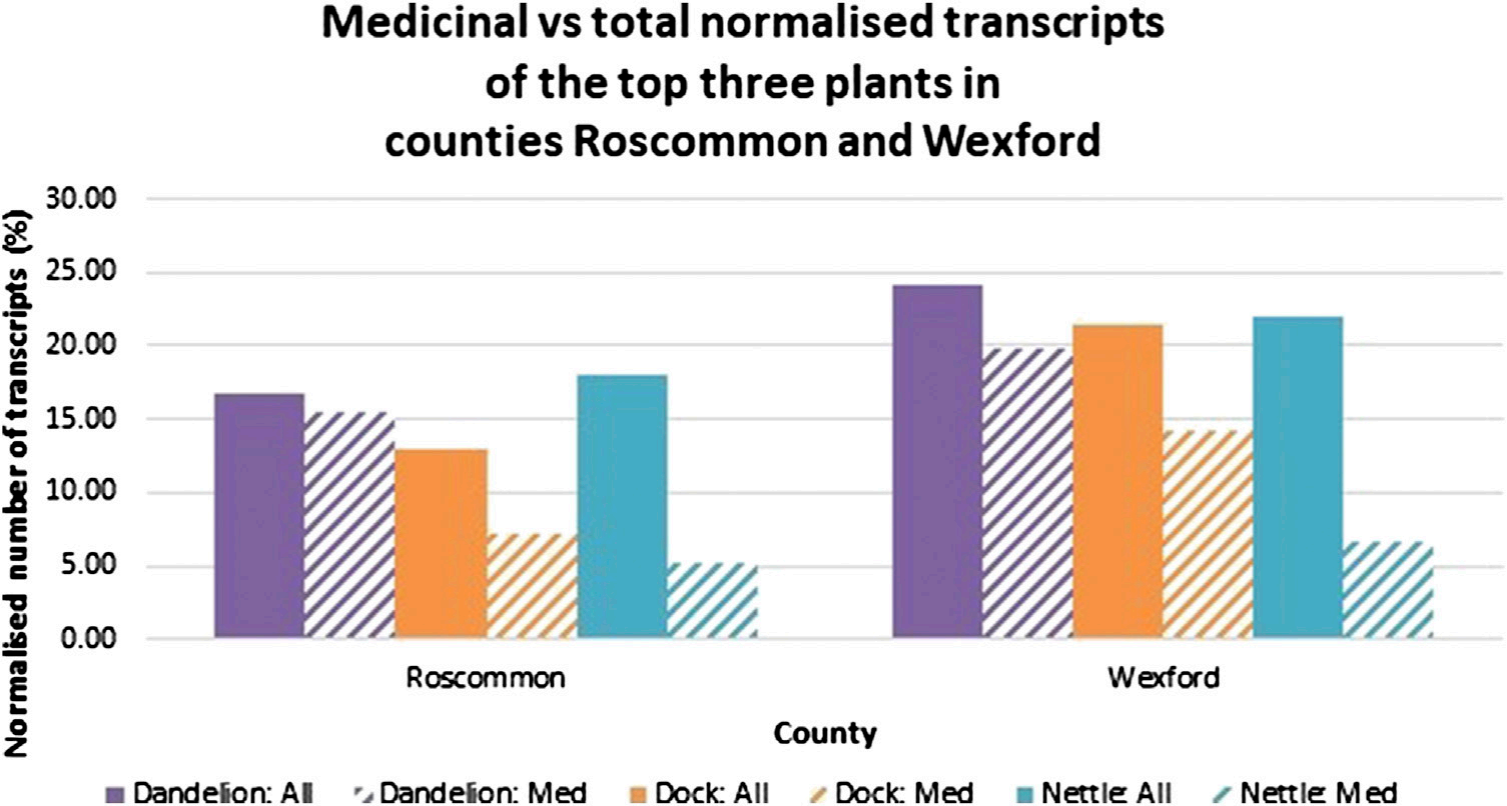
Bar charts contrasting the normalized number of all transcripts (%) of dandelion, dock and nettle with the number of those only relevant to medicinal uses in Roscommon and Wexford. All the retrieved transcripts were scanned manually using the “Find” function of Google Chrome web browser for citations relevant to medicinal uses and those were extracted and counted. The graph was created using Microsoft Excel.

The normalized number of transcripts both unrefined and manually filtered to medicinal uses, citing the top three plants in Roscommon and Wexford are shown in Figure 9. Notwithstanding that the transcripts have been indexed by topic, searching within a topic of interest, such as “folklore medicine,” is not an available function of the digital archive. Therefore, manual data extraction was employed to exclude irrelevant transcripts.

From the data abstracted online, it can be inferred that most citations for dandelions were made in relation to their medicinal uses, especially in Roscommon where almost all transcripts were captured as relevant, supporting its prevalence as a medicinal plant in Ireland in the 1930s. In the case of dock, approximately half the citations in Roscommon and two-thirds in Wexford were related to medicinal uses. Therefore, it can be deduced that dandelion and dock were widely cited in a medicinal context. However, this is not the case for nettle as only a minor proportion of the transcripts were relevant. The contributor to this phenomenon was most of the citations of nettle being made in the context of nettle sting treatment by dock. For instance, when “nettle” was input into the search engine and the results were refined to Wexford, a transcript citing “…The juice of a dock leaf rubbed on a nettle sting eases the pain…” (Vol. 0895, p. 247) was retrieved; this transcript was considered irrelevant as nettle was not cited as valuable for medicinal purposes. Nevertheless, the number of citations of nettle relevant to medicinal uses can be considered significant and it is almost like that of dock in Roscommon. These findings further endorse the status of dandelion, dock and nettle, and the order in which they were ranked, as the top three most frequently cited medicinal plants in Roscommon and Wexford as identified by (Shannon et al., 2018).

Notably, the above transcript would have also been retrieved when “dock” was used as the search word. As such, the citation was successfully captured during the data extraction procedure for dock. Thus, the manual scanning of the retrieved transcripts applied in the data extraction procedure was proven critical in the exclusion of all irrelevant transcripts to refine the dataset. Although the function of full text searching indexed transcripts within a topic of interest would dramatically increase the efficiency of research, manual data exclusion should be considered particularly when it is known that more than one plant of interest is likely to be cited together.

#### Retrievable Detail on Disease Treatment Categories for the Top Three Plants

Disease classification is a challenge in social science research relying on informants. With regard the Dúchas database, such challenges exist due to; the extensive amount of material and many variations of content, the vernacular associated with the era that the material was documented, i.e., 20th century Ireland, and the lack of botanical and or medical expertize held by the children that collected the material. The system is based on two existing classification systems; The International Classification of Primary Care 2nd edition (WICC, 2015), and a system that was established in a 2016 study to analyze a translation of Dioscorides’ De Materia Medica (Staub et al., 2016) (Supplementary Table S1). In this present study, the systematic framework of the disease Classification System effectively aided the data organization process, thereby allowing the extracted information to be analyzed and interpreted rapidly. Notwithstanding that a small number of previously unidentified and ambiguous conditions were encountered during the classification process, the clarity and specificity of the system allowed them to be sorted with ease. This also demonstrates that the classification system is dynamic, as its usage will result in continuous elaboration of the disease classes, leading to the growth in comprehensiveness. Thus, the Shannon et al. (2018) disease classification system was proven to be a highly effective methodical tool in data organization which can be easily adapted and transferred to archival and database research of similar nature. The application of such a system in the research methodology should be encouraged.

**FIGURE 10:**
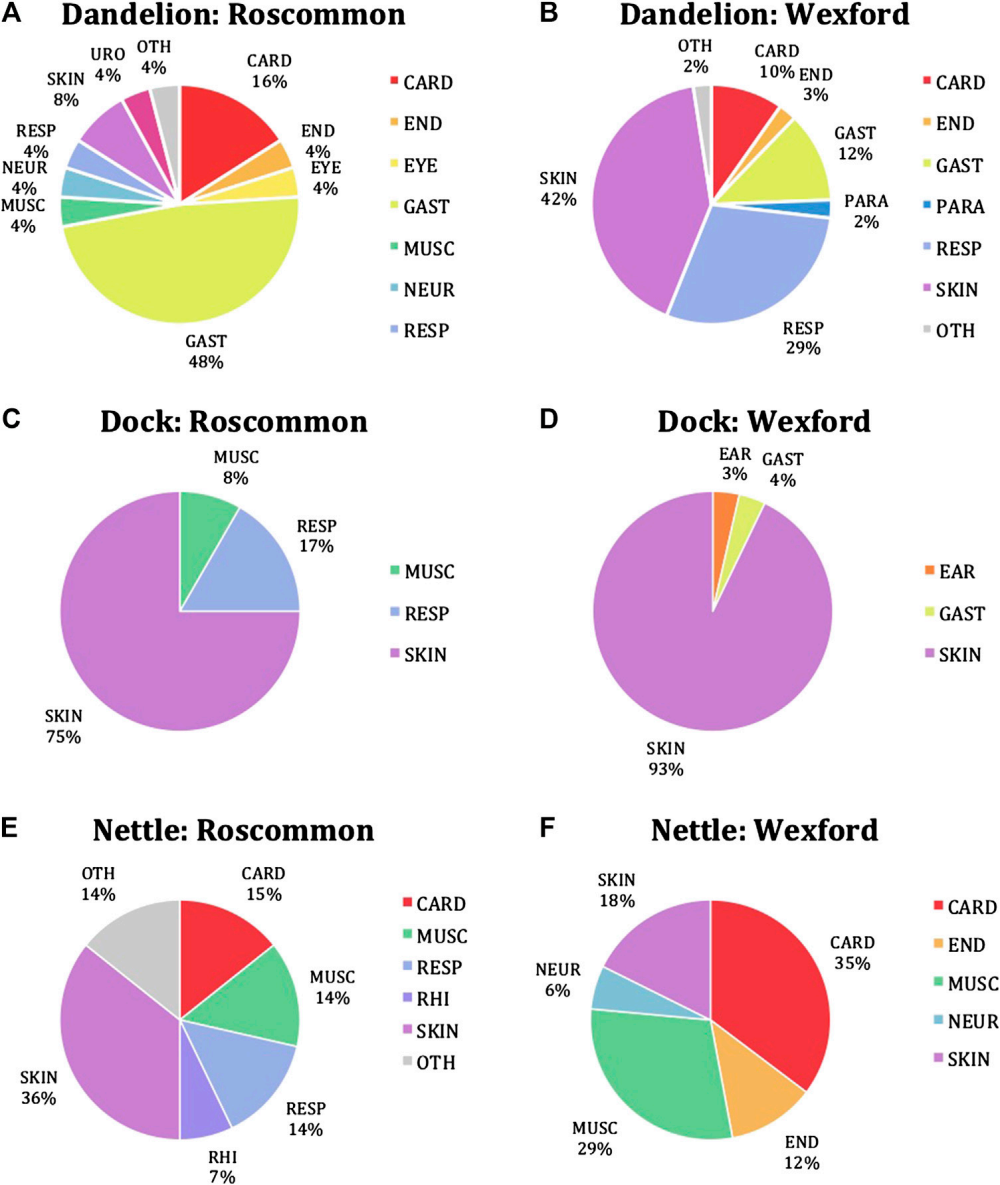
Pie charts showing percentages of cited disease classes for treatment by **(A,B)** dandelion, **(C,D)** dock, and **(E,F)** nettle in Roscommon and Wexford. The disease classification system applied was that established by (Shannon et al., 2018). The pie charts were created using Microsoft Excel.

Dandelion demonstrates the highest diversity in its medicinal uses in the two counties, i.e., eight in Roscommon and seven in Wexford (Figures 10A,B). Moreover, the pie charts depict the heterogeneity in the medicinal uses of Dandelion in the two counties. Approximately half of the citations in Roscommon were related to GAST whereas in Wexford, SKIN was the commonest conditions Dandelion was cited for, making up to 42%. However, only 8% of the citations in Roscommon were made in relation to SKIN treatments and similarly, GAST treatments by Dandelion, making up to 12%, were not as common in Wexford. The second commonest disease group in Wexford is RESP, making up to 29% but only 4% of the citations in Roscommon were relevant to such. CARD treatments were cited in similar frequencies in both counties. Notably, EYE, MUSC, and NEUR, although each cited infrequently at 4%, were exclusive to Roscommon whereas PARA and OTH were only presented in Wexford in relatively minor proportion of 2% each. Analysis of the data shows that GAST is the most highly cited disease category, followed by RESP and then SKIN. Dandelion was most widely used for warts, which falls under SKIN, and as anticipated, such medicinal use was mainly cited in Wexford. Consumption treatment (and prevention) was cited exclusively in Wexford but at a high frequency, so much so that it is the second most frequently cited medicinal use of Dandelion. On the other hand, jaundice, the third commonest medicinal use, was cited only in Roscommon.

For Dock (Figures 10C,D), SKIN is the most highly cited category in both Roscommon and Wexford, representing 75 and 93%, respectively. Dock was almost cited exclusively for SKIN conditions in Wexford, with GAST and EAR accounted for very small fractions of 3 and 4%, respectively. RESP and MUSC each account for a portion of 17 and 8%, respectively in Roscommon. Notably, apart from SKIN, the other disease categories were exclusive to the county in which they were cited. Dock was most widely employed in the treatment for Nettle sing; it accounts for more than half of the SKIN treatments in both counties. Although burn/scorch and sting treatments were ranked second and third, they were cited significantly less frequent than Nettle sting.

From Figures 10E,F it is obvious that the medicinal uses of Nettle in the two counties are heterogeneous. SKIN accounts for the highest number of transcripts in Roscommon at 36% and RHI the lowest at 7%, with CARD, MUSC, RESP, and OTH each with around 14% citation. On the other hand, CARD represents the most significant portion of the pie chart in Wexford, at 35%, followed by MUSC, SKIN, END, and NEUR. CARD, MUSC, and SKIN were the only cited categories common to both counties. Although nettle was most widely cited in the context of SKIN, both blood purification (CARD) and rheumatism (MUSC) represent the most frequent medicinal uses of nettle in both counties. In regard to SKIN, nettle was used the most for pimple treatment. Notably, the roots of Nettle were commonly employed in the treatment of INFEC, particularly measles, in Longford (data not shown).

#### Vernacular Names

To systematically exploit the Dúchas.ie database for ethnomedicinal research, one must be familiar with the vernacular names of the subject(s) of interest relevant to the Irish context in the early 20th and the geographical region(s) of interest, in both English and Gaelic. Although this paper only uses the commonest English vernacular names of the plants of interest in the methodology, Shannon et al. (2017) uncovered that multiple vernacular names have actually been attributed to the plants by students in Counties Wexford and Roscommon in the physical SMC manuscripts. Describes those names for the top three plants in the current paper, namely dandelion, dock, and nettle. Furthermore, the book “Ireland’s Generous Nature” (Jackson, 2014) also lists various common nomenclatures of plants in Ireland. Thus, a rigorously designed methodology should ideally incorporate these vernacular names into the search strategy. Due to the incomplete transcription of the physical SMCs which in turns limits the searchability of the Duchás database, it seems as though the most systematic and rigorous way of compiling the vernacular names of interest would be through thorough examination of the physical SMC manuscripts, in combination with reference sources such as the book “Ireland’s Generous Nature” (Jackson, 2014). However, this would be significantly counterproductive to the advantages and convenience of having an electronic database. As such, resources should be directed toward not only the transcription of the SMCs, but also the compilation of a list of vernacular names of subjects of interest to ethnomedicinal researchers in the SMC. The list should be filterable by factors such as counties and languages. Notably, the complete transcription of the physical SMC could, in fact, also aid the compilation process. Additionally, the incorporation of Boolean operators into the in-built “Find” functionality will also significantly enhance searchability.

### SWOT Analysis of the Dúchas Database and Enthnomedicinal Material Data

**FIGURE 11:**
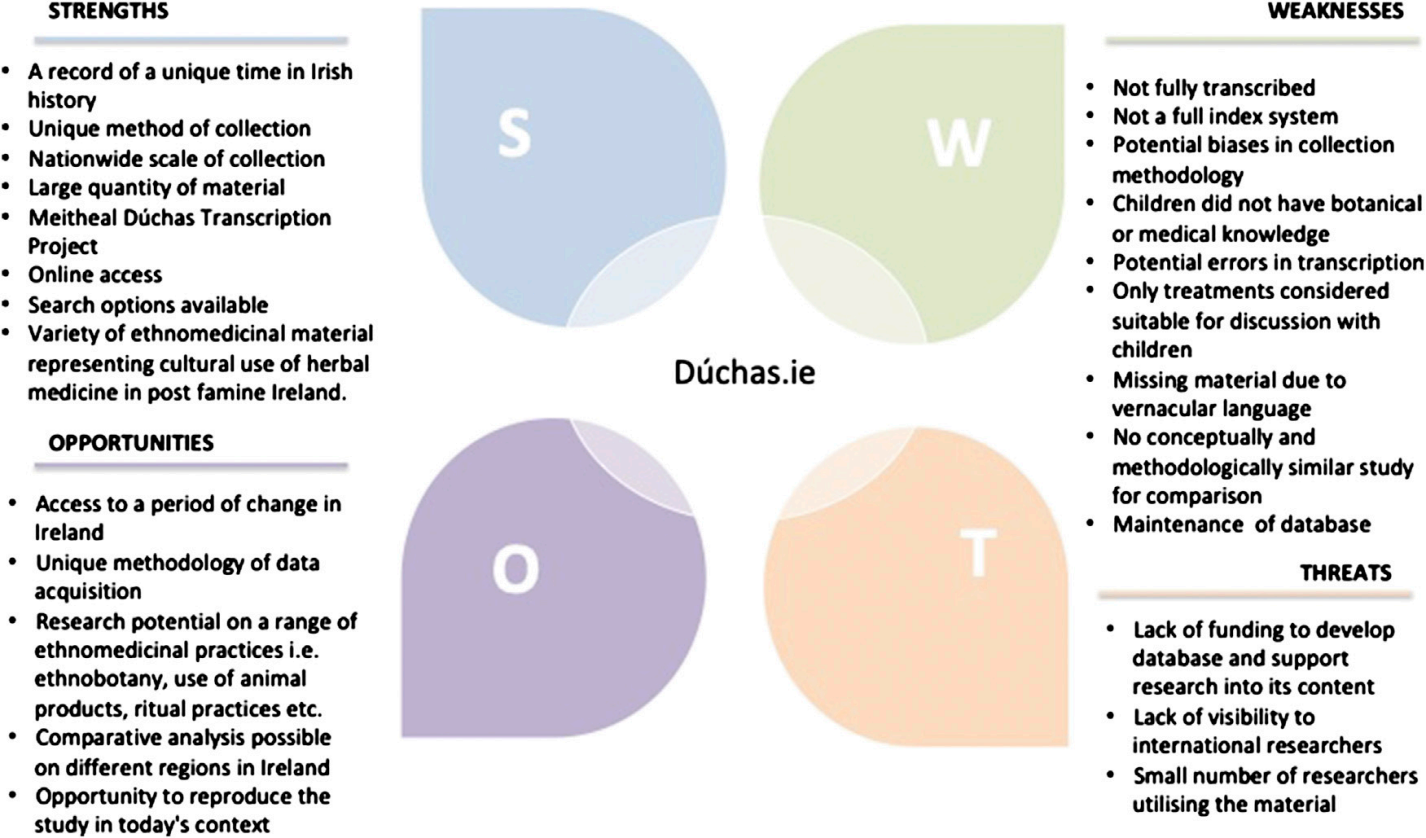
SWOT analysis of the Dúchas.ie database.

A SWOT analysis was carried out which illustrates key strengths, weaknesses, opportunities and threats relevant to the Dúchas database and ethnomedicinal data housed therein (Figure 11).

***Strengths***: The Dúchas database offers a record of a unique time in Irish history, i.e., post-famine and a newly found state. Furthermore, the method of collection was unique and an early example of citizen science that utilized schools, teachers, schoolchildren, and community. The large quantity of material and accessibility through the Meitheal Dúchas Transcription Project are additional strengths, and specifically the variety of ethnomedicinal material representing cultural use of herbal medicine in early 20th century Ireland.

***Weaknesses***: Weaknesses include the previously mentioned potential biases in methodology. These biases are substantiated by the European Citizen Science Association, in the development of the “Ten Principles of Citizen Science,” a framework designed to foster excellence in all aspects of Citizen Science (Robinson et al., 2018). In addition, there is a lack of directly comparative bodies of information, conceptually and methodologically similar to the Dúchas database. The treatments included in database are limited as they were only suitable for children to report. Ireland was a staunchly Catholic country in the 1930s and State and Religion were deeply intertwined (Briody, 2008). Thus, it is not surprising there are no accounts related to gynecological issues, fertility or sexual health. Moreover, the children that collected the material did not have botanical or medical knowledge. Although the whole collection is available online in the form of scanned images, to date, all of the text is not yet transcribed and there is the potential for errors in completed and future transcriptions. In relation to accessing the material, no full index system has been created and it is possible to miss material due to the use of vernacular language. Lastly, the need for maintenance of such a database can be classified as a weakness.

***Opportunities***: The database provides access to a period of change in Ireland, a time when the country was establishing its identity as a free state. It also provides access to material that was acquired through a unique methodology. One of the key opportunities associated with the Dúchas database includes the possibility of reproducing the study in today’s context, in Ireland as well as in other nations. There is potential to carry out comparative analysis on different regions in Ireland, and to specifically explore ethnomedicinal material, that range from ethnobotanical treatments to the use of animal products, religious and ritual practices.

***Threats***: A considerable threat identified is the necessity for funding to develop the database and support research into its content. The Meitheal Dúchas Transcription Project is reliant on volunteers to transcribe the material. In addition, there is a threat of lack global awareness to this ethnographic resource, thus no visibility to international scholars resulting in only a small number of researches utilizing the material.

### Limitations

By focusing on ethnobotanical contributions and utilizing specific plants as “probes,” we illustrate how the database can be used to facilitate modern research and clarify the depth of information that can be obtained on relevant topics. Additionally, we identify potential issues and several limitations associated with the use of Dúchas, and illustrate where possible, methods of counteracting such limitations.

The Meitheal Dúchas Project is not fully transcribed, with 30% of the Gaelic pages and 43% of the English pages completed (8th February 2019). Thus, interpretation of the archive must be conducted with care This limitation will be attenuated over time. The normalization procedure applied in this present study also successfully counteracts such limitations.

Many of the transcripts Dates on the material supplied were not routinely and systematically extracted by transcribers. Therefore, this is a limitation which represents an opportunity for the data transcribing and systemization process to be improved.

Transcripts, although classified into different topics, cannot be searched by a specific term within a topic of interest, such as “folk medicine.” Therefore, the retrieved transcripts from full-text searching comprise citations in contexts other than medicinal uses and the volume of the retrieved transcripts prevented manual exclusion of irrelevant transcripts. Such functionality would enable more research opportunities and enhance the effectiveness and efficiency of archival research.

The in-built “Find” function of the database can retrieve transcripts containing the search term(s) entered. When a single term, e.g., “tea,” is entered, a huge volume of transcripts is retrieved. However, if more than one term is entered, the database only retrieves transcripts with the exact terms in the exact order. For instance, searching “**tea cure**” will retrieve “Fresh strawberry leaves made into **tea cure** sore mouths and throats,” but not “A **cure** for sore eyes is to wash them with cold **tea** without any milk or (hot) sugar and they will get alright” since the two terms are apart in the transcript.

The sole usage of the commonest English vernacular name of each plant of interest, to the exclusion of others, in data extraction represents another limitation, because citations of the plants in other synonyms or in other languages such as Gaelic would not have been captured and included in the analysis.

The accuracy of the number of retrieved citations is based on the accuracy of the transcription. Typographical mistakes, e.g., “dandelion” instead of “dandelion,” made by those transcribing would result in retrieval failure, leading to incomplete data extraction and therefore inaccurate data analysis and interpretation. Nevertheless, such risk can be mitigated by the accessible transcript-editing and error-reporting system in place on the website. A small number of retrieved transcripts showed very similar or the same wordings of information and therefore appeared to be transcription duplicates. Examination of the sources of transcripts and the handwriting revealed that different students produced them. Considering that and the fact that the risk presented to the integrity of the data is insignificant, they were not excluded from the study.

The importance of the quality of citizen science research has been addressed in recent years, with the development of “The Ten Principles” (Robinson et al., 2018). As the information on the Dúchas database predates this framework, the integrity, reliability and quality of the records in the archive might be of concern as the scheme intrinsically lacked quality assurance due to the nature of collection. As discussed before, the parties involved in the collection process might not have employed high level of precision, particularly the schoolchildren who were responsible for data recording. Nonetheless, the methodology in this present study counteracted such limitation by appropriate data normalization. Such limitation is perhaps commonly associated with archival research since the data of interest might have been collected without high emphasis on accuracy. Thus, policies in maintaining the integrity and quality of data in any currently on-going or future archival or database construction are important, and greater emphasis on the data collection process will ensure them to be reliable research sources.

## Conclusion

For the first time this study explores in detail the use of the unique database Dúchas.ie, an online archive that contains over half a million pages of ethnographic material, collected in an early example of citizen science, by school children in 1930s Ireland.

Dúchas.ie is a valuable resource, offering one of the first tangible examples of Citizen Science in operation, and providing a unique insight into post-famine Ireland. Although conducting research on an incomplete electronic database does come with its own limitations, the Dúchas.ie database carries tremendous research potential that waits to be realized. One should also bear in mind that although ethnographic research can provide rich insights that would otherwise be impossible, it is invariably confounded by various uncontrollable uncertainties regardless of the methodology and resources of choice. Attention and resources should be directed to the enhancement of the Dúchas.ie database, a true national treasure of Ireland, to encourage, enable and empower accessibility and open science in Irish ethnomedicinal research in the 21st century.

## Data Availability Statement

The raw data supporting the conclusions of this article will be made available by the authors, without undue reservation.

## Author Contributions

All authors contributed to the paper. The Research was directed and supervised by HS, AS, and MH. The research and writing was carried out by AK and FS.

## Conflict of Interest

The authors declare that the research was conducted in the absence of any commercial or financial relationships that could be construed as a potential conflict of interest.
