# Supplementary material for: Exploring the Irish National Folklore Ethnography Database (Dúchas) for Open Data Research on Traditional Medicine Use in Post-Famine Ireland: An Early Example of Citizen Science
Source: Front Pharmacol. 2020 Oct 29;11:584595. doi: 10.3389/fphar.2020.584595 (PMC7723452; doi:10.3389/fphar.2020.584595)
Supplement: Supplementary file 1 [file Table1_v1.DOCX]

| **Disease Classification**  **Categories** | | **International Classification of Primary Care (ICPC) 2^nd^ Edition (1998) – listed using the ICPC defined subcategories** | **Lenoti et al (2016) - classification based on data translated from Dioscorides’ De Materia Medica**  **(ex Matthioli, 1568)** | **The Schools’ Manuscript Collection Disease Classification System generated by Shannon *et al.*, 2018** |
| --- | --- | --- | --- | --- |
| Organs | *Eyes* | *Categorised as Eye:*  Symptoms (pain, discharge..); infections (conjunctivitis..); neoplasm; injuries; congenital anomalies; other diagnosis (glaucoma, blindness, retinopathy..) | *Categorised as Ophthalmology:*  Eye problems | ***Categorised as Eye (EYE):***  Cataract, eye disease, eyesight problems (syn), inflamed eyes, object in eye (syn), sore eye (syn), sty (syn), wart on eye (syn) |
|  | *Ears* | *Categorised as Ear:*  Symptoms (pain, ache, tinnitus, bleeding hearing..); Infections; neoplasm; injuries (foreign body in ear..); congenital anomalies; other diagnosis (excessive wax, deafness, acoustic trauma..) | *Categorised as Otology:*  Ear problems | ***Categorised as Ear (EAR):***  Deafness, earache, pain in ear (syn) |
|  | *Skin* | *Categorised as Skin:*  Symptoms (skin pain, lumps/swelling, rash, colour change..); infections (herpes zoster/simples, scabies, impetigo..); neoplasms; injuries (animal bite, sting, bruise, cut..); congenital anomalies (moles..); other diagnosis (urticaria, psoriasis, cyst, eczema, acne..) | *Categorised as Dermatology:*  Ulcers, abscesses, animal stings and bites, burns, tumours, gangrene, fistula, skin inflammation, indurations, carbuncles, chilblains, pustules dandruff | ***Categorised as Skin (SKIN):***  Animal bite (syn), bleeding (syn), blister (syn), boil (syn), bruise (syn), burn (syn), carbuncle, chapped lips, chapped hands, chilblains, cold sore, corn, cut (syn), drawing poison, eczema, erysipelas (syn), frost bite, hives, insect sting (syn), itch, nettle sting (syn), pimples, rash, ringworm (syn), scratch, shingles, skin complexion, skin troubles (syn), sores (syn) sore lips, sunburn, swelling (syn), thorn, ulcers, warts, whitlow (syn), wrinkles |
| Systems | *Musculoskeletal* | *Categorised as Musculoskeletal:*  Symptoms (neck, back, arm chest, wrist, shoulder complaint..); infections; neoplasm; injuries (fractures, sprain, dislocation..); congenital anomalies; other diagnosis (bursitis, rheumatoid arthritis, osteoporosis, tennis elbow..) | *Categorised as Musculoskeletal ailments:*  Cramps, fractures, gout, hematoma and spasms | ***Categorised as Musculoskeletal (MUSC):***  Backache (syn), bone breast out of place (syn), bracket shins, broken bone, bunions, cramps (syn), disease of the bone, feet issues (syn), fracture, gout, joint pain (syn), lame girl, rheumatism (syn), sciatica, spasm, sprain (syn), swollen joints (syn), weak spine |
|  | *Digestive*   - *Parasites* - *Oral* | *Categorised as Digestive:*  Symptoms (abdominal cramps, heartburn, nausea, vomiting, diarrhoea..) infections (gastrointestinal infections, mumps, viral hepatitis..); neoplasm (stomach, colon/rectum, pancreas..); injuries; congenital anomalies; other diagnosis (teeth, gum disease, mouth/ tongue/ lip disease, liver diseases, appendicitis, peptic ulcer, ulcerative colitis, worms/other parasites..) | *Categorised as Gastroenterology:* Diarrhoea, dysentery, spleen afflictions, jaundice, abdominal pain and stomach ache, liver problems, flatulence, hiccup, hematemesis, vomiting  *Categorised as Parasites:*  Lice, scabies, tapeworms  *Categorised as Oral Cavity:* Dentistry, gums and tonsils | ***Categorised as Gastroenterology (GAST):***  Antibilous tonic (syn), bad stomach (syn), bowel complaints (syn), colic, constipation, diarrhoea, digestion (syn), dyspepsia (syn), dysentery, heartburn, hiccough (syn), jaundice/yellow jaundice, liver complaints (syn), pit of the stomach down (syn), sea sickness, stomach complaints (syn), swallowed something (syn), vomiting  ***Categorised as Parasites (PARA):*** Worms, worm fever, tail worms  ***Categorised as Oral (ORAL):***  Bad tooth (syn), bleeding from pulled tooth, fallen palate (syn), gumboil, gums, sore mouth, thrush (syn), tonsils, toothache (syn), whiten teeth |
|  | *Cardiovascular and Blood* | *Categorised as Cardiovascular:*  Symptoms (heart pain pressure, tightness, swollen ankles, palpitations..); infections (rheumatic fever..); neoplasm; congenital anomalies; other diagnosis (Ischaemic heart disease, heart failure, heart disease, high blood pressure, haemorrhoids, varicose veins, stroke..)  *Categorised as Blood, Blood Forming Organs and Immune Mechanism:*  Symptoms (blood symptom/complaint, lymph glands enlarged/ painful..); infections (lymphadenitis acute..); neoplasm (leukaemia, Hodgkin’s disease..); injuries (ruptured spleen..) congenital anomalies; other diagnosis (Anaemia, blood/lymph/spleen disease..) | *Categorised as Cardiovascular:* Haemorrhoids, varicose veins, cardiac chest pain, vasodilation, blood liquefier | ***Categorised as Cardiovascular and Blood Conditions (CARD):***  Bad heart, blood, bloodless person, blood disease (syn) blood medicine, blood poison, blood pressure, blood purifier (syn), blood tonic, heart, heart disease, heart fever, heart problem (syn) heart stimulant, heart tonic, internal bleeding, internal bruises and spitting up blood, piles, varicose veins |
|  | *Respiratory*   - *Rhinology* | Categorised as Respiratory:  Symptoms (nosebleed/ epistaxis, nose symptom/complaint, pain in respiratory system, shortness of breath, wheezing, cough, sneezing..); infections (whooping cough, strep throat, influenza, pneumonia, pleurisy, conjunctivitis..); neoplasm; injuries; congenital anomalies; other diagnosis (asthma, chronic obstructive pulmonary disease, allergic rhinitis..) | *Categorised as Respiratory:*  Asthma, coughs, dyspnoea, tuberculosis  *Categorised as Rhinology:*  Epistaxis and polyps | ***Categorised as Respiratory (RESP):*** Asthma, bronchitis, catarrh, chest complaints (syn), cold (syn), cough (syn), croup (syn), hoarseness, lung complaints (syn), pleurisy, pulmonary fever, quinsy, scrofula, sore throat, tuberculosis (syn), whooping cough (syn)  ***Categorised as Rhinology (RHI):***  Nose bleeds (syn) |
|  | *Urological* | *Categorised as Urological:*  Symptoms (painful urination, ); infections (conjunctivitis..); neoplasm; injuries; congenital anomalies; other diagnosis (glaucoma, blindness, retinopathy..) | *Categorised as Urology:* | ***Categorised as Urological (URO):*** Backache with kidney disease, bladder complaints (syn), brights disease, dropsy, kidney complaints (syn) |
|  | *Neurological*   - *Psychological* | *Categorised as Neurological:*  Symptoms (headache, restless legs, convulsion/ seizure, vertigo/dizziness..); infections (poliomyelitis, tetanus..); neoplasm; injuries (concussions, head injury); congenital anomalies; other diagnosis (multiple sclerosis, epilepsy, parkinsonism, migraine..)  *Categorised as Psychological:*  Symptoms (feeling anxious, sleep disturbances, feeling depressed..) other diagnosis (dementia, schizophrenia, affective psychosis, suicide attempt, post traumatic stress disorder..) | Categorised as Neurology:  Including psychosomatic ailments | ***Categorised as Neurological and psychological (NEUR):***  Asleep foot, convulsions, encephalitis (syn), epilepsy (syn), insanity, light head (syn), nerves (syn), neuralgia, nightmares, palsy, sleeplessness |
|  | *Endocrine, Metabolic and Nutritional* | *Categorised as Endocrine, Metabolic and Nutritional:*  Symptoms (excessive thirst, appetite, loss of appetite, weight gain/loss..); infections (endocrine infection); neoplasm (of the thyroid); congenital anomalies (thyroglossal duct/cyst..); other diagnosis (goitre, gout, obesity, vitamin/nutritional deficiency, hypoglycaemia, diabetes..) | *Categorised as Food:*  Including spices | ***Categorised as Endocrine, Metabolic and Nutritional (END):***  Appetite issues (syn), builder, diabetes, drink, excess weight, famine food, food, goitre, nourishment, rickets, scurvy, strength, tonic (syn), weak hair |
|  | *Reproductive*  *(Including male and female)* | *Categorised as Pregnancy, Childbearing, Family planning:*  Symptoms (question of pregnancy, contraception..); infections (puerperal infection/ sepsis..); neoplasm (related to pregnancy); injuries (during pregnancy); congenital anomalies (complication during pregnancy); other diagnosis (ectopic pregnancy, gestational diabetes, stillbirth..)  *Categorised as Female Genital:*  Symptoms (genital pain, menstrual pain, menstruation irregularity..)  .); Infections (syphilis female, gonorrhoea, genital herpes, chlamydia..); neoplasm (of cervix, breast..); injuries; congenital anomalies; other diagnosis (vaginitis, cervical disease..)  *Categorised as Male Genital:*  Symptoms (pain in penis/testis/scrotum, urethral discharge, prostate symptom..); infections (syphilis male, gonorrhoea, genital herpes..); neoplasm; injuries; congenital anomalies (undescended testicle); other diagnosis (benign prostatic hypertrophy, genital disease male..) | *Categorised as Andrology:*  Male fertility  *Categorised as Gynaecology:*  Female fertility | ***Categorised as Reproductive systems and conditions (REPRO):***  Female, women’s problems, syphilis (syn) |
| General and  Un-  specified | *Cancer* |  |  | ***Categorised as Cancer (CAN):***  Cancer |
|  | *Esoteric* |  |  | ***Categorised as Esoteric (ESO):***  Evil eye, fairy children, fortune teller/nurse, protection from fairies, witches and wizards, restore friendships, superstition |
|  | *Fevers* |  |  | ***Categorised as Fever (FEV):***  Fever (syn) |
|  | *Infectious diseases* |  |  | ***Categorised as Infectious Disease (INFEC):***  Chickenpox (syn), diphtheria, flu (syn), measles, mumps, scarlet fever |
|  | *Antidotes and poisons* |  | *Categorised as Antidotes:*  *Categorised as Poisons:*  Hunting and fishing poisons | ***Categorised as Antidote (ANT):***  Poison |
|  | *Preventative* |  |  | ***Categorised as Preventative Treatment (PREV):***  A preventative treatment for any condition |
|  | *Other* | *Categorised as General and Unspecified:*  Symptoms (Pain, chills, fever, fainting, swelling..); Infections (tuberculosis, measles, chickenpox, malaria, rubella..); neoplasms (unspecified location); Injuries (unspecified trauma/ injury; congenital anomaly (not specified); other diagnosis | *Categorised as Others:*  Magic, indefinable pathologies, unspecific inflammations, veterinary medicine and uses related to humeral pathology without any link to specific organs. | ***Categorised as Others (OTH):***  Ailments (syn), apoplexy, argue, Australian ache, baldness (syn), cures (syn), deaf and dumb, delicate (syn), disabled people, drowning, drowsy child, good health (syn), guts and bladder, infection, induces perspiration, inflammation, inward growth, pain (syn), rupture (syn); vernacular conditions that were not identified (blood spine ailments,, duoreasc, evil, irac, ireac, ireach, sore beats, tálac, thrallac, the hig) |
